# A Novel Design of a Plate for Posterolateral Tibial Plateau Fractures Through Traditional Anterolateral Approach

**DOI:** 10.1038/s41598-018-34818-5

**Published:** 2018-11-06

**Authors:** Dong Ren, Yueju Liu, Jian Lu, Runtao Xu, Pengcheng Wang

**Affiliations:** Orthopaedic Trauma Service Center, Third Hospital of Hebei Medical University, Major Laboratory of Orthopaedic Biomechanics in Hebei Province, Shijiazhuang, Hebei Province China

## Abstract

Biomechanical performance of a newly designed plate for treating posterolateral tibial plateau fractures was compared with three traditional internal fixation devices using finite element analysis (FEA) and biomechanical experiments. Forty synthetic tibias were used to create posterolateral shearing tibial fracture models, which were randomly assigned to groups A–D. The fragments were fixed with two 6.5-mm lag screws (group A), the newly designed plate (group B), a 3.5-mm lateral locking plate (group C), and a posterolateral buttress plate (group D). In the biomechanical experiment, vertical displacement of the posterolateral fragments was measured under axial loads of 500–1500 N. In the FEA, vertical displacement of the posterolateral fragments and stress distribution and maximum stress of each internal fixation were measured under axial loads of 250–750 N. Biomechanically, collective ranges of vertical displacements in the four groups were 0.356 ± 0.089–1.055 ± 0.023 mm at 500 N axial load, 0.651 ± 0.062–1.525 ± 0.03 mm at 1000 N, and 0.903 ± 0.077–1.796 ± 0.04 mm at 1500 N. Differences between the four groups were statistically significant (P < 0.05), except for groups B and C at 1500 N. FEA showed that collective ranges of vertical displacements in the four groups were 0.290–1.425 mm at of 250 N axial load, 0.580–1.680 mm at 500 N, 1.067–1.818 mm at 750 N. Maximum stress of groups A–D were, respectively, 321.940, 132.660, 100.383, and 321.940 MPa under 250 N axial load. Maximum stress of all four internal fixations increased, and the overall trends at 500 and 750 N were consistent with that at 250 N. Posterior, straight fixation was the most reliable. Fixation with the lag screw was least reliable. The new plate and 3.5-mm lateral locking plate exhibited similar control over fragment displacement. The newly designed plate was stable and reliable, indicating its suitability for clinical application.

## Introduction

Posterolateral tibial plateau fractures account for about 8–15% of all tibial plateau fractures. The mechanism of this fracture is a combination of valgus stress and axial compression forces with the knee in flexion, the bone intensity of the lateral femoral condyle is higher than the lateral condyle of the tibial plateau, thereby causing the fracture^1^. Because of its unique anatomy, surgical treatment of the posterolateral tibial plateau fracture is challenging,there is no consensus on the treatment strategy. To fully expose and reset these fractures, most studies have focused on the surgical approach, including the anterolateral, posteromedial, fibular osteotomy, and improved posterolateral approaches^2–9^. These surgical approaches, however, may greatly further damage the fracture, produce a poor surgical field, cause fixation difficulty, and provide insufficient stability of the knee joint because of delayed repair of the lateral knee joint capsule and iliotibial tract.

**Figure 1:**
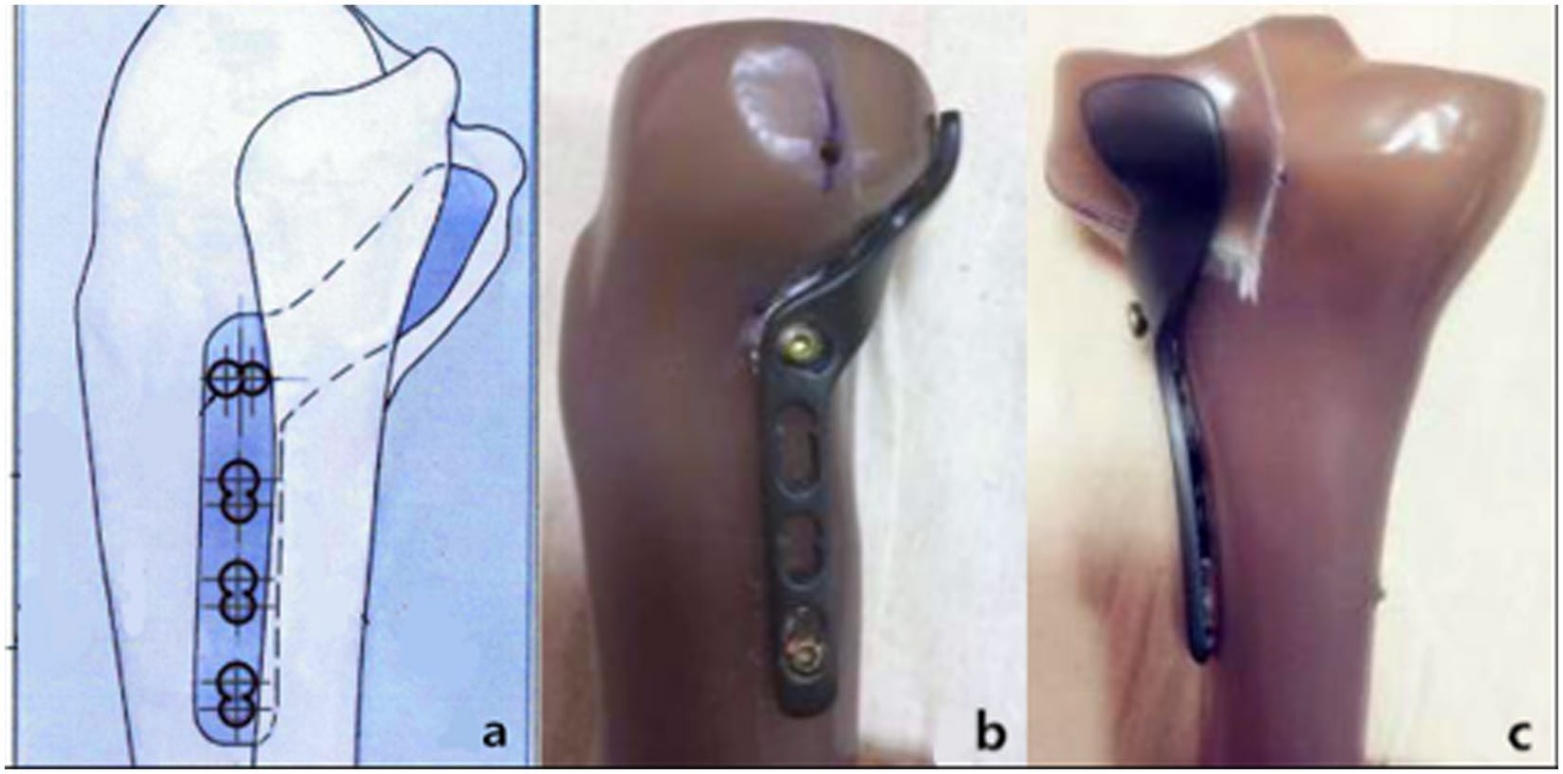
Newly Designed Plate for Fixation of Posterolateral Tibial Plateau Fractures.

To solve this problem, we previously performed an anatomical study to determine the spatial structure of the proximal tibia–fibula joint. Based on the results, we developed a novel plate (Fig. 1) for posterolateral tibial plateau fractures and applied for a patent (Patent No. CN104706413A). The purpose of the present study was to compare the biomechanical performance of this novel plate with those of three conventional internal fixation methods currently in use. We aimed to prove its stability and provide a rationale for its use in fixing posterolateral tibial plateau fractures.

## Materials and Methods

Forty synthetic left tibias (type 3401; Sawbones AG, Vashon Island, WA, USA) were used to create posterolateral shearing tibias. The newly designed plates (Shandong Weigao Medical Instrument Co., Ltd., Zibo, China), 6.5-mm lag screws, 3.5-mm lateral locking plates, and 3.5-mm six-hole posterolateral buttress plates (Depuy Synthes,Eimattstrasse 3,4436 Oberdorf,Switzerland) were used to fix the fractures in the models. The Electroforce 3520-AT electronic universal material testing machine ((TA Instruments, New Castle, DE, USA) was used to test the biological mechanics of these systems. Vertical displacement of the posterolateral fragments was measured using a laser displacement sensor with a precision of 0.001 mm (HG-C1030; Panasonic Industrial Devices SUNX Suzhou Co., Ltd., Ayutthaya, Thailand).

## Modeling and Groups

**Figure 2:**
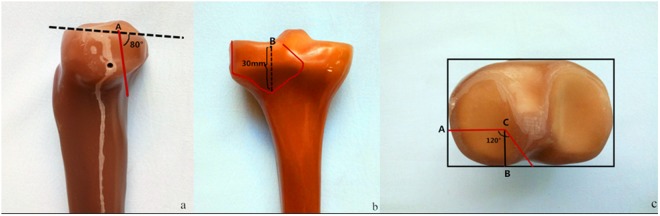
Model of a Posterolateral Tibial Platform Fracture.

According to previous research on posterolateral tibial plateau fractures^10^, Forty synthetic tibias were used to make posterolateral shearing tibial fracture models (Fig. 2) and randomly assigned to one of four groups (A–D, 10 per group). Each group was fixed with different implants.

Group A: Lag screw technique, wherein two anterolateral 6.5-mm parallel lag screws were placed at 10 and 20 mm, respectively, from the lateral edge and 5 mm below the joint line.

Group B: The newly designed plate, which was placed underneath the fibula capitulum anterolaterally, with the oblique part of the plate adjoined to the posterolateral fragments inferoposteriorly with no screws inserted. At first, a screw was inserted in the distal screw hole, then another one was inserted through the sliding hole located at the junction of the oblique and straight sections, that reduced and compacted the fragments further by sliding the plate superoanteriorly. Two consecutive locking screws were then inserted in the remaining holes.

Group C: A 3.5-mm, L-shaped, proximal tibia, lateral anatomic locking plate. After inserting four proximal locking screws parallel to the articular surface, we inserted four consecutive locking screws in the four distal screw holes.

**Figure 3:**
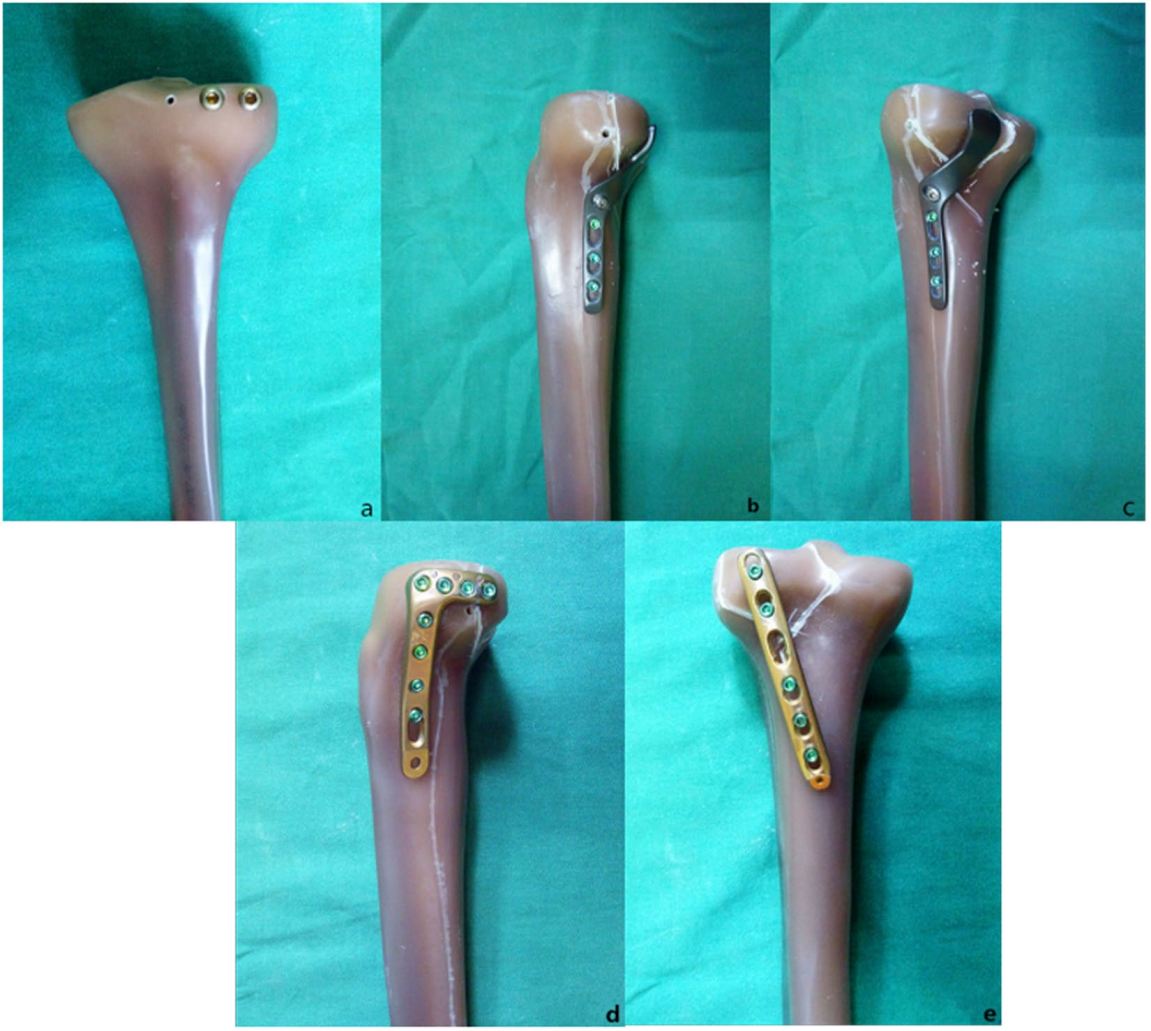
Implant types. (**a**) Two anterolateral 6.5-mm parallel lag screws, (**b,c**) Newly designed plate. (**d**) A 3.5-mm lateral anatomic locking plate. (**e**) A 3.5-mm six-hole posterolateral buttress plate.

Group D: A posterolateral 3.5-mm six-hole straight buttress plate was placed obliquely (proximal from the lateral aspect and distal from the medial aspect) without molding. Two cancellous screws were placed in the posterolateral fragment, and three cortical screws were inserted distally (Fig. 3).

One orthopedic surgeon reduced and fixed each fracture according to standard operating procedures.

### Finite element analysis

Specimens from each group were randomly extracted and then scanned using 64-slice CT with the main scan parameters as follows: tube voltage 120 kV, tube current 200 mA, thickness 1 mm, index 1 mm. All image data were saved in Digital Imaging and Communication in Medicine format (resulting in 866 data images).

**Figure 4:**
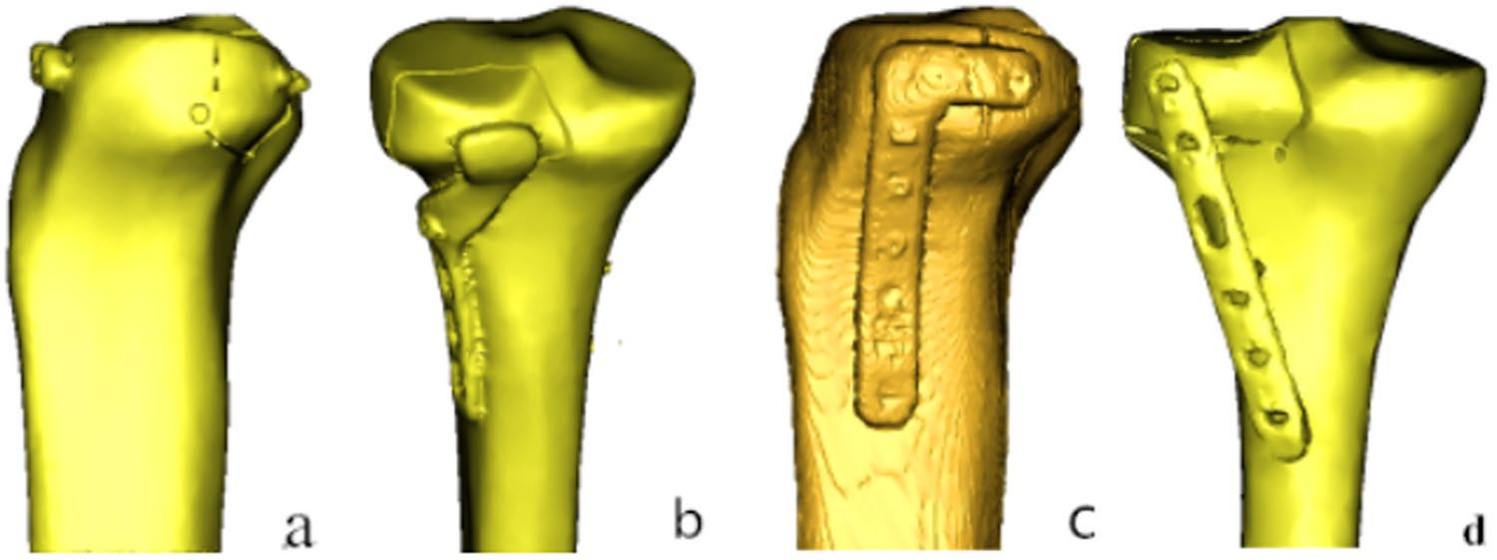
Three-dimensional Models of the Four Groups.

The CT images were then imported into an interactive medical image control system (Mimics 10.01; Materialise, Leuven, Belgium) for pretreatment and three-dimensional (3D) reconstruction. The preliminary reconstructed 3D images were divided into surface meshes by Mimics’ Magics 9.9 rigid-lattice division program. The 3D models were optimized by the finite element analysis (FEA) module of Mimics software along with creation of surface meshes. The surface mesh models were converted into volume mesh models with the Mesh tool of the ANSYS preprocessor (ANSYS, Inc., Canonsburg, PA, USA). The models of the volume meshes were re-imported into Mimics software to endow material properties. The elastic modulus and Poisson ratio of each portion was selected according to the literature^11,12^. Elastic moduli of 14,000 and 200,000 MPa were chosen for the tibia and implants, respectively. Poisson’s ratio was assigned as 0.40 and 0.30 for the tibia and implants, respectively. The numbers of elements and nodes of various models in the experiment are shown in Table 1. The models are shown in Fig. 4.

**Table 1:** Number of Nodes and elements for the Four Models in the Experiment.

Model	Nodes	Elements
Group A	420000	230000
Group B	294000	166000
Group C	184000	100000
Group D	474000	272000

The models were then imported into ANSYS for set loading and boundary conditions. First, to set the 3D axis of the tibia, the line that intersects the coronal and horizontal tibial plateau planes is defined as the X axis; the line that intersects the horizontal and sagittal planes is defined as the Y axis; and the line that intersects the coronal and sagittal planes is defined as the Z axis. In this study, the tibia model, internal fixation, and screws are designed to be continuous, homogeneous, and of isotropic linear elastic material. In addition, the translation and rotation of the distal tibial underside was constrained in three dimensions.

The literature shows that the posterolateral tibial plateau bears the largest load when the knee joint is flexed 90°. Verifying the stability of the fixation is the main target of our research, so the load on the posterolateral fragments were defined as 250, 500, and 750 N^10,13^, with the loading direction parallel to the Z axis of the tibial plateau.

Malreduction has been defined as an intra-articular gap of 2 mm, which is a figure we also used. In this experiment, we ignored the factors influencing knee stress, including ligaments, muscles, and other soft tissue. We analyzed vertical displacement of the posterolateral fragments and the stress distribution and maximum stress of each internal fixation under axial loads.

### Biomechanical testing

**Figure 5:**
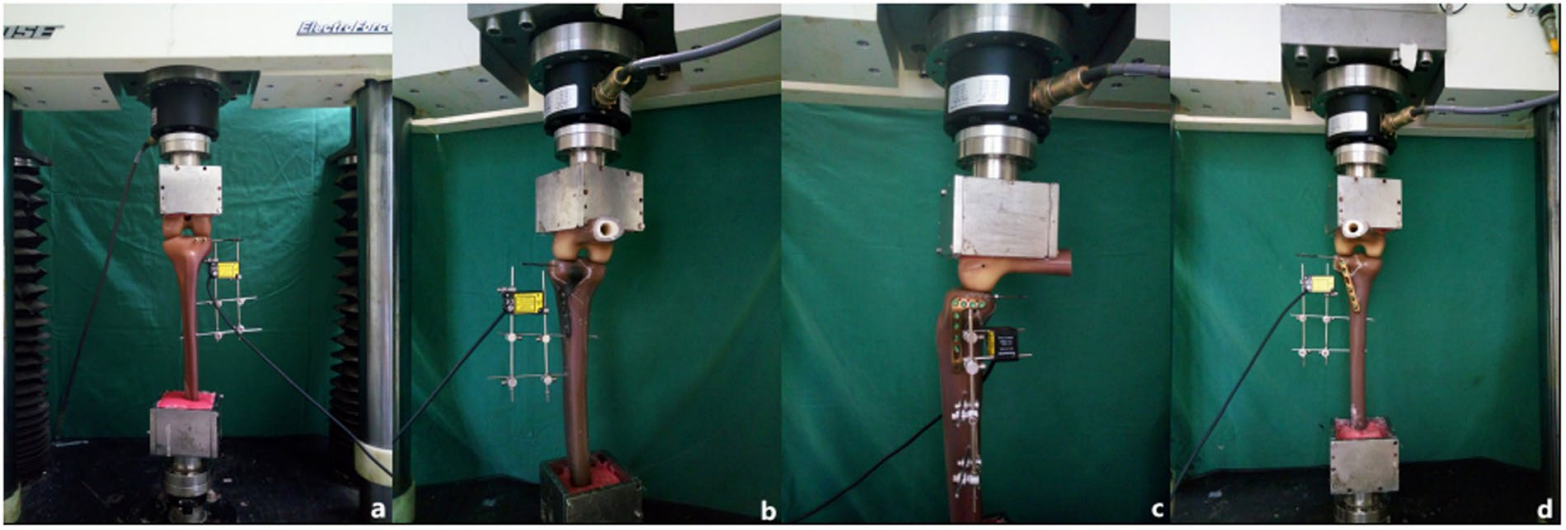
Biomechanical Experimental Machine. The vertical displacements of the posterolateral fragments were measured using a laser displacement sensor with an inserted K-wire.

A synthetic femur (left) (type 3403; Sawbones AG) was used as an applicator to deliver axial forces on both tibial plateau surfaces. The distal femur was fixed in 90° flexion to simulate the injury mechanism (an axial force with the knee in flexion). A tailor-made K-wire was inserted into the posterolateral fragment (but not exceeding the fracture line) (Fig. 5). Vertical displacement of the posterolateral fragment was then measured using a laser displacement sensor and the measurement exported to special software. A compressive force was applied with a loading speed of 10 N/s. Axial peak loads of 500, 1000, and 1500 N were chosen. During a pilot study, fixation failure, or screw loosening, did not occur up to 1500 N, displacements of the posterolateral fragment never reached 2 mm, so we did not set the final load until fixation failure.

### Statistical analysis

As the data were normally distributed, we used parametric statistical methods for data analysis. Descriptive statistics were used to determine ranges, means, and standard deviations. Analysis of variance was used for data from the four groups under the same load. If P < 0.05, a least-significant difference (LSD) post hoc multiple comparison was used for the vertical displacement data. Data were analyzed using SPSS 19.0 Statistical Software (SPSS Inc., Chicago, IL, USA). A value of P < 0.05 was considered to indicate statistical significance.

## Results

### FEA

**Figure 6:**
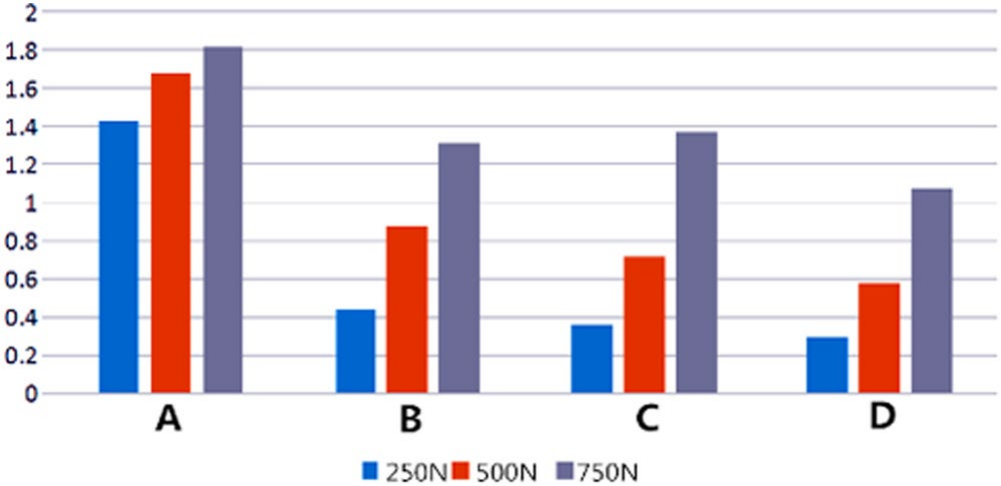
Vertical Displacement of the Posterolateral Fragments of the Four Groups Under Different Loads. (**A**) Two anterolateral 6.5-mm parallel lag screws (**B**) Newly designed plate. (**C**) A 3.5-mm lateral anatomic locking plate. (**D**) A 3.5-mm, six-hole posterolateral buttress plate

**Figure 7:**
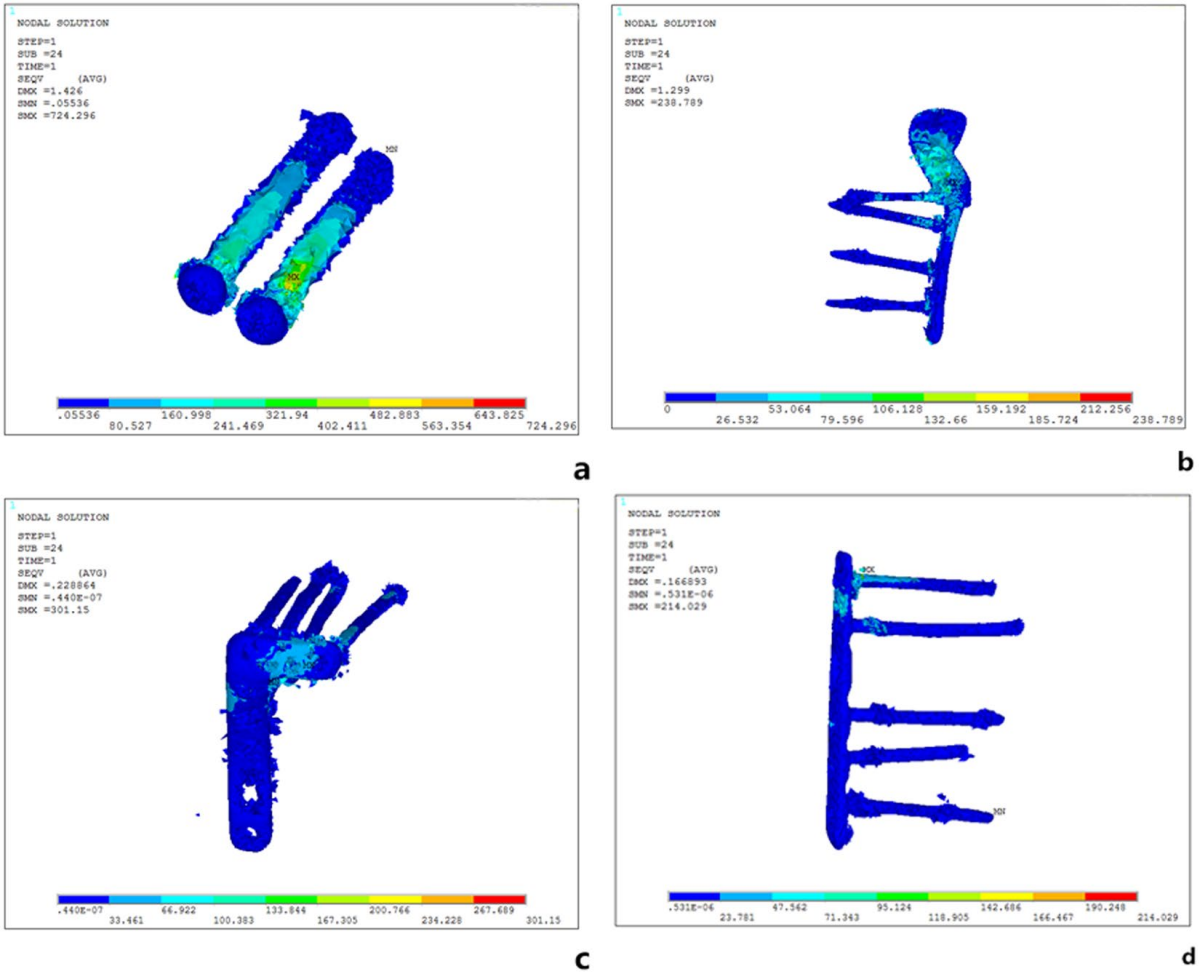
Stress Distribution of the Four Groups Under an Axial Load of 250 N. (**a**) Group A: the stress was mainly focused on tail closing to the end cap; (**b**) Group B: the stress was mainly focused on the junction of the oblique and straight portions and the two proximal screws. (**c**) Group C: the stress distribution was more uniform, with the increase found on local areas of the head’s side and corner and on the screw located most posteriorly. (**d**) Group D: the stress was mainly focused on the tails of two proximal screws and the local area of the plate between them.

Vertical displacements of the posterolateral fragments in each of the four groups gradually increased under loads from 250 N to 750 N (Fig. 6). In order of the largest to smallest displacements under loads of 250 N, the methods were rated as follows: group A-B- C-D. The overall trends when the axial loads were 500 N and 750 N were consistent with the 250 N findings. Whereas displacement of the posterolateral fragments in each of the four groups reflects their stability intuitively, the stress distribution and maximum stress of each internal fixation reflect it indirectly. Stress associated with the two anterolateral 6.5-mm lag-screw technique was mainly focused on tail closing to the end cap under loads of 250 N, with the strong stress concentration there (Fig. 7a). The stress of the newly designed plate was mainly focused on the junction of the oblique and straight portions and the two proximal screws (Fig. 7b). The lateral locking plate stress distribution was more uniform, with the increase found on local areas of the head’s side and corner and on the screw located most posteriorly (Fig. 7c). The stress associated with the posterolateral buttress plate was mainly focused on the tails of two proximal screws and the local area of the plate between them (Fig. 7d). The maximum stress of groups A, B, C, and D were, respectively, 321.940, 132.660, 100.383, and 71.343 MPa under an axial load of 250 N. Maximum stress increased in all four internal fixation techniques when the load increased, when the axial loads were 500 N and 750 N, the stress distribution was consistent with that of 250 N (Table 2).

**Table 2:** Maximum Stress Experienced by Internal Fixation Devices Under Various Axial Loads, by Group.

	Maximum stress (Mpa)		
	250 N	500 N	750 N
Group A	321.940	479.551	565.267
Group B	132.660	262.988	393.308
Group C	100.383	200.305	317.57
Group D	71.343	145.617	264.711

### Biomechanical testing

Vertical displacement of the posterolateral fragment under three axial loads is summarized in Table 3. In order, from the largest to the smallest displacements under loads of 500 N it was A-B-C-D; under loads of 1000 N it was A-B-C-D; under loads of 1500 N it was A-C-B-D. Except for groups B and C under 1500 N, the differences between the four groups were statistically significant (P < 0.05).

**Table 3:** Vertical Displacement of the Posterolateral Fragment Under Various Axial Loads, by Group.

Groups	Vertical displacement (mm)		
	500 N	1000 N	1500 N
A	1.055 ± 0.023	1.525 ± 0.035	1.796 ± 0.04
B	0.543 ± 0.049^a^	0.901 ± 0.064^a^	1.269 ± 0.073^a^
C	0.379 ± 0.024^ab^	0.744 ± 0.049^ab^	1.321 ± 0.052^a^
D	0.356 ± 0.089^abc^	0.651 ± 0.062^abc^	0.903 ± 0.077^abc^
P value	0.000	0.000	0.000
F value	333.76	376.60	120.65

^a^Compared with group A, *P* < 0.05; ^b^compared with group B, *P* < 0.05; ^c^compared with group C, *P* < 0.05.

## Discussion

### Clinical significance of biomechanical experiments

This study was based on an FEA and an experimental biomechanical method. We compared the biomechanical properties of a newly designed plate with three traditional internal fixation for posterolateral tibial plateau fractures. The results of the study showed that the maximum displacement after using these four internal fixation techniques under vertical pressure of 1500 N was less than 2 mm. It was found that the four treatments for tibial plateau fractures were effective and feasible.

In addition, the FEA showed stress concentrations in each of the four internal fixation techniques. The stress was greatest using the anterolateral 6.5-mm parallel lag screws and smallest with the straight buttress plate. Stress experienced with the new plate and the lateral locking plate was similar. The greater maximum stress concentrates, the more likely fracture pieces display again at the initiation of postoperative functional exercise, even another operation was required^14^. The stability of our new plate is similar to that of the lateral locking plate.

In all, our newly designed plate was stable and reliable, indicating its suitability for clinical application.

### Current treatment for posterolateral fracture of the tibial plateau

Posterolateral tibial plateau fracture has attracted the attention of an increasing number of authors, who have provided different resolutions for it. Solomon *et al*. described treatment using fibula neck osteotomy that allowed anterolateral and posterolateral exposure, with subsequent fixation with a plate anteriorly or laterally. The follow-up results were significantly better than those using the anterolateral approach, although it could lead to injury of the common peroneal nerve or nonunion^7^. Frosch *et al*. presented a surgical approach that involves lateral arthrotomy for visualizing the joint surface and a posterolateral approach for fracture reduction and plate fixation, both of which are achieved through one posterolateral skin incision, with good curative effect^15^. The Tscherne–Johnson extensile approach to tibial plateau fractures increases direct visualization of the lateral plateau articular fractures and protects IT band insertion.^16^. Carlson reported a patient who had bicondylar fractures of the tibial plateau^17^ and in whom exterior and interior incisions of the knee joint were used along with a double plate.

Although surgical exposure to the tibial plateau via an anterolateral approach is easy, the lack of wide exposure and inadequate reduction of the fracture can be a problem. Also, it is difficult to achieve strong internal fixation with anterolateral lag screws and an anterolateral plate. Access through a posterior exposure is wider and achieves strong internal fixation. Surgeons must have both perfect technique and anatomical knowledge of the popliteal fossa region because the access is adjacent to important vessels and nerves. Thus, effective approaches are available, but all have some limitations. There is no consensus about the approach or the choice of internal fixation for posterolateral fractures of the tibial plateau.

### Advantages of the new plate for posterolateral tibial plateau fractures

We found that traditional approaches were unable to provide full visualization, interfering with adequate fixation of the fractures. Or itself may injures the vessels and nerves, and many other shortcomings might be envisioned. Based on these findings, we devised a palm-shaped plate that could be used to embrace the fracture fragments. We then measured the anatomical space around the tibia–fibula joint and used designed a new plate for posterolateral tibial plateau fractures.

Technically, the new plate is composed of a straight and an inclined plate, both of which are rectangular. The inclined plate is connected to the top of the straight plate. The slanting angle of the inclined plate is matched to the angle of the tibia’s lateral surface. Then, two to four screw holes are distributed straight along the plate. The newly designed plate has the advantage of causing less damage during the operation. This new plate is placed via a traditional anterolateral incision, which is less damaging and is convenient. The straight plate is fixed to the tibia using screws. The oblique plate avoids exposure of the fibula, posterior lateral complex, anterior tibial artery, and other important anatomical structures that could influence fixation of the fracture–greatly reducing the possibility of damage caused by the surgery.

Compared with the traditional operation, our new plate has many advantages. (1) It can be implanted through traditional anterolateral approach,that maintaining the stability of the posterolateral structures without damage, and avoiding nearby important structures. While implantation is simple. It reduces bleeding and can shorten the operation time. The supine position is an easy placement, and the results of intraoperative fluoroscopy are easily evaluated. (2) The newly designed plate proved to have good mechanical properties, and its head was similar to that of the anti-slide buttress plate. (3) It did not affect the treatment of concomitant medial and the anterior lateral fractures. (4) If necessary, removal of internal fixation is relatively simple. (5) For comminuted fractures, Fixing the fragments with screws is difficult. The new plate, however,can holds the comminuted fragments like a “palm” —a good solution to this problem.

### limitations of the study

Though artificial tibial model has been widely used in biomechanics research, yet it differs from fresh frozen specimen in bone density, compression deformation, and fractional friction coefficients. Also, the fracture we simulated is a simple one that did not take into account tissues around the knee joint, including the ligaments, meniscus, nerves, blood vessels, and other factors that may affect the actual outcome^14,18,19^. All of these factors need to be further explored in the future.

### Research prospects and future clinical applications

Our research may provide a new option in the treatment of posterolateral tibial plateau fractures. So far, we have measured the anatomical space of the proximal tibiofibular joint for designing the new plate. FEA and biomechanics research have been undertaken to determine the reliability of the new plate. A clinical series will be conducted to provide a dependable basis for the clinical application of this new treatment option for posterolateral tibial plateau fractures.
